# Improved Reconstruction of *In Silico* Gene Regulatory Networks by Integrating Knockout and Perturbation Data

**DOI:** 10.1371/journal.pone.0008121

**Published:** 2010-01-26

**Authors:** Kevin Y. Yip, Roger P. Alexander, Koon-Kiu Yan, Mark Gerstein

**Affiliations:** 1 Department of Computer Science, Yale University, New Haven, Connecticut, United States of America; 2 Department of Molecular Biophysics and Biochemistry, Yale University, New Haven, Connecticut, United States of America; 3 Program in Computational Biology and Bioinformatics, Yale University, New Haven, Connecticut, United States of America; University of Manchester, United Kingdom

## Abstract

We performed computational reconstruction of the *in silico* gene regulatory networks in the DREAM3 Challenges. Our task was to learn the networks from two types of data, namely gene expression profiles in deletion strains (the ‘deletion data’) and time series trajectories of gene expression after some initial perturbation (the ‘perturbation data’). In the course of developing the prediction method, we observed that the two types of data contained different and complementary information about the underlying network. In particular, deletion data allow for the detection of direct regulatory activities with strong responses upon the deletion of the regulator while perturbation data provide richer information for the identification of weaker and more complex types of regulation. We applied different techniques to learn the regulation from the two types of data. For deletion data, we learned a noise model to distinguish real signals from random fluctuations using an iterative method. For perturbation data, we used differential equations to model the change of expression levels of a gene along the trajectories due to the regulation of other genes. We tried different models, and combined their predictions. The final predictions were obtained by merging the results from the two types of data. A comparison with the actual regulatory networks suggests that our approach is effective for networks with a range of different sizes. The success of the approach demonstrates the importance of integrating heterogeneous data in network reconstruction.

## Introduction

The expression of genes is tightly controlled by the regulatory machinery in the cell. A major part of which involves regulator proteins such as transcription factors (TFs). Transcription regulation can be modeled as a directed network with each node representing a gene and the proteins that it encodes, and an edge from one node to another if the former is a regulator of the latter. In addition to the directionality, the edges are also signed, with a positive sign indicating a positive regulation (activation) and a negative sign indicating a negative regulation (suppression).

Methods have been proposed for computationally reconstructing regulatory networks. One common approach is to use differential equations to model how the expression levels of genes change according to the abundance of their regulator proteins over time [1]–[4]. Since it has only recently been possible to quantitatively measure the abundance of proteins in each cell for many proteins simultaneously by flow cytometry [5], protein abundance has long been approximated in two ways: 1) the expression level of mRNA has been used as a proxy of the quantity of the corresponding protein; 2) a multi-cell average has been used as a proxy of the quantity in individual cells. With the use of mRNA level to approximate protein abundance, both the data for estimating the expression level of a gene and the activity of its regulators are obtained from the same mRNA microarray assays. Each set of experiments involves an initial experimental condition (e.g., an environmental perturbation such as a heat shock), which affects the expression levels of some genes that react to the condition. Expression profiles are then obtained at different time points as a measure of the changing internal state of the cell.

In the resulting dataset, each data point measures the expression level of a gene in a specific condition at a certain time point. Each such observed value is determined by a mixture of different factors, including the previous expression level of the gene, the activity of its regulators, decay of mRNA transcripts, randomness, and measurement errors. The many entangled parameters make it difficult to reconstruct the regulatory network based on this type of data alone.

To decode this kind of complex systems, one strategy is to reduce it to a series of subsystems with manageable sizes by keeping the values of most parameters constant and varying only a small number of them. Thanks to the creation of large-scale deletion libraries [6], it is now possible to carry out this divide-and-conquer approach. A deletion library contains different strains of a species (e.g. yeast), each of which has one of the genes of the species disabled – completely (*knocked out*) by mutagenesis [6] or partially (*knocked down*) by RNA inference (RNAi) [7]. Profiling the expression of each gene in a deletion strain allows one to study the sub-network that is affected by the deleted gene. For instance, if the deleted gene encodes for a protein that is the only activator of another gene, then the expression level of the latter would be dramatically decreased in the deletion strain of the former as compared to the wild-type strain in which the regulator gene is intact.

Sophisticated computational methods have been developed in previous studies to use deletion data to infer regulatory networks. For example, Bayesian approaches have been used to model biological pathways and the effects of gene deletion [8]. Factor graphs have been used to model protein-protein and protein-DNA interactions. The maximum a posteriori values of the parameters, learned by a message-passing belief propagation procedure, can be used to explain observed data and infer pathway memberships [9], [10]. Other probabilistic models have also been used to infer proteins that are upstream of others in the regulatory cascade [11].

While deletion data is good for detecting simple, direct regulatory events, they may not be sufficient for decoding those that are more complicated. For example, if a gene is up-regulated by two TFs in the form of an OR circuit, so that the gene is expressed as long as one of the TFs is active, these edges in the regulatory network cannot be uncovered by single-gene deletion data. In such a scenario, traditional time course data could supplement the deletion data in detecting the missing edges. For instance, if at a certain time point both the TFs have a low abundance and the expression rate of the gene is observed to be impaired, this observation could potentially help reconstruct the OR circuit.

As another example, if a regulator is normally not expressed, deleting its gene would not cause an observable effect to the expression of other genes. Yet if in a certain perturbation the expression of the regulator is induced by the external stimuli, its regulation of other genes could be detected.

Therefore, the two types of data are complementary in reconstructing regulatory networks. In this study we demonstrate how they can be used in combination to improve network reconstruction. We first propose methods for predicting regulatory edges from each type of data, and then describe a meta-method for combining their predictions. Using a set of fifteen benchmark datasets, we show the effectiveness of our approach, which led our team to get the first place in the public challenge of the third Dialogue for Reverse Engineering Assessments and Methods (DREAM) [12], [13], “a concerted effort by computational and experimental biologists to understand the limitations and to enhance the strengths of the efforts to reverse engineer cellular networks from high-throughput data” [14]. We will also discuss potential weaknesses of our approach, and directions for future studies.

## Methods

### Problem Definition

We first formally define our problem of reconstructing regulatory networks. The target network is a directed network with 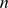 nodes. The edges are completely unobserved, and we are to predict them from the data features alone. In other words, this is an unsupervised learning setting. The edges are signed, but these signs are not considered in our experimental evaluation. The goal is thus to learn a model from the data features, such that given an ordered pair of two genes 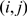, it can predict whether 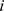 is a regulator of 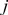.

We use two types of data features: perturbation time series data and deletion data. Deletion data are further sub-divided into homozygous deletion and heterozygous deletion.

In a perturbation time series dataset, an initial perturbation is performed at time 0, which sets the expression levels of each gene to a certain level. Then the regulatory system is allowed to adjust the internal state of the cell by up- and down-regulating genes according to the abundance of the TFs. The expression level of each gene is taken at subsequent time points. Thus, for each perturbation experiment, each gene is associated with a vector of real numbers that correspond to its expression level at different time points after the initial perturbation. If there are 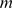 perturbation experiments and the 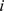-th one involves 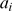 time points, then each gene is associated with a vector of 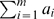 expression values.

In a deletion dataset, a gene is deleted, and the resulting expression level of each gene at steady state is measured. By deleting each gene one by one, and adding the wild-type (no deletion) as control, each gene is associated with a vector of 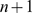 values, corresponding to its steady-state expression level in the 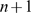 strains. For diploid organisms (with two copies of each gene in the genome), the deletion can be homozygous (with both copies deleted, i.e., “null mutant”) or heterozygous (with only one copy deleted).

We assume that both types of deletion data, as well as perturbation data, are available, although it is trivial to modify our algorithm by simply removing the corresponding subroutines if any type of data is missing.

### The Learning Method

Our basic strategy is to learn the simple regulation cases from deletion data by using noise models, and to learn the more complex ones from perturbation data using differential equation models. We first describe the two kinds of models and how we learn the parameter values from data, then discuss our way to combine the two lists of predicted edges into a final list of predictions.

#### Learning noise models from deletion data

We consider a simple noise model for deletion data, that each data point is the superposition of the real signal and a reasonably small Gaussian noise independent of the gene and the time point. The Gaussian noise models the random nature of the biological system and the measurement error. Based on this model, the larger is the change of expression of gene 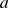 from wild type to the deletion strain of gene 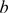, the more unlikely that the deviation is due to the Gaussian noise only, and thus the larger chance that 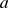 is directly or indirectly regulated by 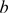.

Notice that the regulation could be direct (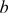 regulates 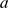) or indirect (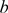 regulates 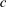 that directly or indirectly regulates 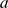). There are studies that try to separate the direct regulation from the indirect ones using methods such as graph algorithms [15] and conditional correlation analysis [16]. In this study we do not attempt to distinguish direct and indirect regulation, and show that even assuming all significant deviation in deletion data to be direct regulation could already provide substantial performance improvements over approaches that focus on perturbation data only.

Given the observed expression level 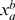 of a gene 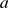 in the deletion strain of gene 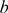, and its real expression level in wild type, 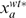, we would like to know whether the deviation 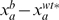 is merely due to noise. To answer this question, we would need to know the variance 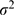 of the Gaussian, assuming the noise is non-systematic and thus the mean 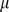 is zero. If the value of 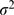 was known, then the probability for observing a deviation as large as 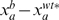 due to random chance only would simply be 
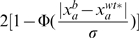
, where 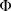 is the cumulative distribution function of the standard Gaussian distribution. The complement, 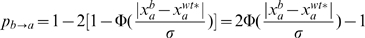, is the probability that the deviation is due to a regulation event. One can then rank all the gene pairs 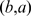 in descending order of 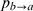.

To implement the above procedure, it is necessary to estimate 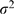 from data, which is standardly done by using the non-biased sample variance of data points that are not affected by the deleted gene. However, this involves two difficulties. First, the set of genes not affected by the deleted gene is unknown and is exactly what we are trying to learn from the data. Second, the observed expression value of a gene in the wild-type strain, 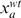, is also subjected to random noise, and thus cannot be used as the gold-standard reference point 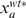 in the calculations.

We propose an iterative procedure to progressively refine our estimation of 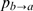. We start by assuming the observed wild-type expression levels 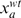 are reasonable rough estimates of the real wild type expression levels 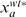. For each gene 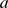, our initial estimate for the variance of the Gaussian noise is set as the sample variance of all the expression values of 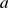 in the different deletion strains. Using them as the initial reference points, we repeat the following three steps for a number of iterations:

1. Calculate the probability of regulation 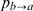 for each pair of genes 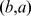 based on the current reference points 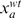. Then use a p-value of 0.05 to define the set of potential regulation: if the probability for the observed deviation from wild type of a gene 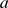 in a deletion strain 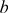 to be due to random chance only is less than 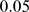, we treat 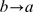 as a potential regulation. Otherwise, we add 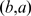 to the set 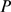 of gene pairs for refining the error model.
2. Use the set 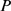 to re-estimate the variance of the Gaussian noise, 
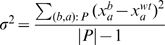
.
3. For each gene 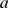, we re-estimate its wild-type expression level by the mean of its observed expression levels in strains in which the expression level of 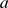 is unaffected by the deletion: 
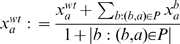
.

After the iterations, the probability of regulation 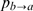 is computed using the final estimate of the reference points 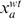 and the variance of the Gaussian noise 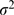.

Notice that we have chosen to use a “conservative” p-value of 0.05 in the following sense: when the number of genes in the network, 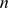, is sufficiently large (e.g. 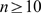) and there are relatively few regulatory edges, there is a large number of gene pairs for estimating the parameters such that missing some of them would not seriously affect the estimation. It would thus be good to add to 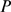 only gene pairs that are very unlikely to contain regulatory edges, so that we would not miss the few real regulatory events. This is achieved by using a large (i.e., conservative in this context) p-value to define the potential regulatory edges.

The above iterative procedure can be applied to both homozygous and heterozygous deletion data, although the regulation signals are expected to be less clear in the heterozygous case since deleting only one copy of a regulator gene may induce only a mild effect to its targets. The final p-values computed from homozygous data are thus expected to be more reliable. Yet the ones learned from heterozygous data can still be useful references in resolving ambiguous cases, as we will discuss in more detail when describing our approach to combining the predictions learned from the different types of data.

Comparing to previous methods, our approach to using deletion data in inferring regulatory events is relatively simple. On the one hand, this is to cope with the limited types of data provided in the DREAM challenge. For instance, direct binding data is not available, and thus cannot be used to setup prior distributions for parameter values, as in some previous studies [9]. On the other hand, instead of having a goal of modeling a whole network or sub-network, our main objective in this study is to identify the most likely regulatory events in the network, which is a simpler and more manageable task in the current setting. While our method works well, as to be shown in the results section, we acknowledge that when trying to obtain a deep understanding of the detailed regulatory mechanisms, richer computational models are needed.

#### Learning differential equation models from perturbation time series data

For time series data after an initial perturbation, we use differential equations to model the gene expression rates. The general form is as follows:
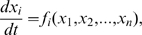
(1)where 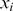 represents the expression level of gene 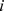 and 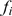 is a function that explains how the expression rate of gene 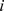 is affected by the expression level of all the genes in the network, including the level of gene 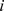 itself. Various types of function 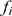 have been proposed. We consider two of them. The first one is a linear model [2]:
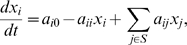
(2)where 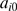 is the basal expression rate of gene 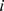 in the absence of regulators, 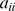 is the decay rate of the mRNA transcripts of 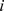, and 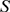 is the set of potential regulators of 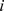 (in this study we assume no self-regulation, so 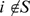). In theory, 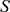 could be set as 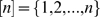, i.e., the whole set of genes in the network, as the regulators of 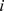 are unknown. However, there are two main issues for such full models, namely the need of an unfeasibly large number of data points for learning the parameter values, and the excessive computation requirement. Therefore, we choose to restrict 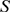 to some small sets, the details of which will be discussed below. For each potential regulator 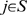, 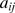 explains how the expression of 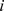 is affected by the abundance of 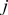. A positive 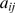 indicates that 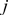 is an activator of 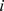, and a negative 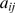 indicates that 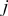 is a suppressor of 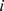.

The linear model assumes a linear relationship between the expression level of the regulators and the resulting expression rate of the target. It is a rough first approximation of the expression rate. An advantage of it is the small number of parameters (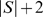), yet real biological regulatory systems seem to exhibit non-linear characteristics. The second model we consider assumes a sigmoidal relationship between the regulators and the target [4]:
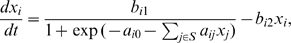
(3)where 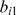 is the maximum expression rate of 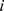 and 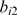 is its decay rate. This model involves 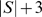 parameters.

Our goal is to try different possible regulator sets 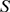 and identify the ones that predict the observed expression levels well in the least-square sense:
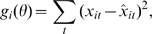
(4)where 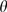 denotes the set of parameters (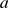
's, 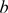
's and 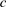
's), 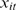 is the expression level of gene 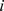 at time point 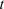, and 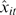 is the corresponding predicted level of a model. The summation is taken over all time points of all perturbation experiments.

The objective function is not convex with respect to the parameters. We use Newton's method [17] to find local minima of the objective function 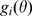 with 100 random initial values of 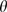, and adopt the one that provides the best fit with the smallest 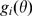. The expression vector 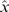, gradient 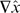 and Hessian 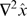 are estimated by using the closed-form formulas provided by the second order Runge-Kutta method [18].

We try two types of regulator sets 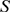. The first type involves single regulators, in which we try each gene 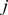 as the potential regulator of gene 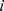 in turn, and compare the least square errors of their best-fit models. The second type involves high-confidence potential regulators, plus one extra regulator to be tested. As we will see in the next section, the high-confidence potential regulators are obtained from the predictions of the noise models learned from the deletion data, as well as those predicted by the single-regulator differential equation models. We call such models the “guided models” since the construction of the regulator sets is guided by previous predictions. The full detail of the resulting algorithm will be given in the next subsection.

We also tried double regulator sets with all pairs of potential regulators. Yet the resulting models did not appear to provide much additional information on top of the single regulator set models, while requiring much longer computational time. We therefore decided to consider only the single regulator sets and guided single regulator sets.

For a regulator set 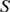 and a target gene 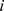, the value of the objective function of the best model indicates how likely 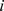 is regulated by the members of 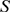. The values are thus used to rank the likelihood of existence of the regulatory edges.

#### Combining the predictions of the models

Our main idea for combining the predictions of the different models learned from deletion and perturbation data is to rank the predictions according to our confidence that they are correct. Specifically, we make predictions in batches, with the first batch containing the most confident predictions, and each subsequent batch containing the most confident predictions that have not been covered by the previous batches. Within each batch, the predictions are ordered by the confidence of the models, which corresponds to the probability of regulation 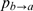 for noise models, and negated objective score 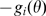 for differential equation models. We define the batches as follows:

- Batch 1: all predictions with a probability of regulation larger than 0.99 according to the noise model learned from homozygous deletion data
- Batch 2: all predictions with an objective score two standard deviations below the average according to all types (linear AND sigmoidal) of differential equation models learned from perturbation data
- Batch 3: all predictions with an objective score two standard deviations below the average according to all types of guided differential equation models learned from perturbation data, where the regulator sets contain regulators predicted in the previous batches, plus one extra potential regulator
- Batch 4: as in batch 2, but requiring the predictions to be made by only one type (linear OR sigmoidal) of the differential equation models as opposed to all of them
- Batch 5: as in batch 3, but requiring the predictions to be made by only one type of the differential equation models as opposed to all of them
- Batch 6: all predictions with a probability of regulation larger than 0.95 according to both the noise models learned from homozygous and heterozygous deletion data, and have the same edge sign predicted by both models
- Batch 7: all remaining gene pairs, with their ranks within the batch determined by their probability of regulation according to the noise model learned from homozygous deletion data

In general, we put the greatest confidence in the noise model learned from homozygous deletion data as the signals from this kind of data are clearest among the three types of data. We are also more confident with predictions that are consistently made, either by the different types of differential equation models (batches 2 and 3 over batches 4 and 5) or by the noise models learned from homozygous and heterozygous deletion data (batch 6).

## Results

### Datasets and Performance Metrics

We used the algorithm described above to take part in the third Dialogue for Reverse Engineering Assessments and Methods Challenge (DREAM3) [19] on *in silico* regulatory network reconstruction, provided by Marbach et al. [20]. It involves fifteen benchmark datasets, five of which have 10 genes, five have 50 and five have 100. The structures of the benchmark networks were obtained by extracting modules from real biological networks [20]. At each size, two of the networks were extracted from the regulatory network of E. coli, and three were extracted from yeast.

The predictions are compared against the actual edges in the networks by the DREAM organizer using four different metrics for evaluating the accuracy:

- AUPR: The area under the precision-recall curve
- AUROC: The area under the receiver-operator characteristics curve
- pAUPR: The p-value of AUPR based on the distribution of AUPR values in 100,000 random network link permutations
- pAUROC: The p-value of AUROC based on the distribution of AUROC values in 100,000 random network link permutations

While the statistics related to the ROC curve are commonly used to evaluate prediction results, those related to the PR curve could be more sensitive when there is a much larger negative set than positive set.

These metrics are further aggregated into an overall p-value for each size using the geometric mean of the five p-values from the five networks, and finally an overall score equal 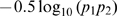, where 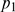 and 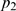 are the geometric means of pAUPR and pAUROC respectively.

In the evaluation by the DREAM organizer, edge signs (activation vs. suppression) are not considered. We note that our algorithm can actually detect edge signs. In the noise model, a regulation is determined as an activation if the resulting expression is higher than the estimated wild-type expression, and a suppression otherwise. For different equation models, a positive sign of the coefficient 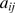 indicates that 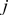 is an activator of 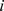, and a negative sign indicates that 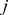 is a suppressor of 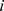.

### Prediction Performance

The challenge of size 10 has attracted 29 teams to participate, the one of size 50 has 27 teams and the one of size 100 has 22 teams. The large number of participants makes the challenge currently the largest benchmark for gene network reverse engineering [19].

Our algorithm ended up in first place on all three network sizes. The complete set of performance scores for all teams can be found at the DREAM3 web site [19]. Below we summarize our prediction results, and discuss some interesting observations.

Table 1 and Table 2 show the AUROC and pAUROC values of our predictions reported by the DREAM organizer, respectively. From the p-values, we see that our predictions are consistently significantly better than random. In general, we observe that our method relatively unaffected by the network size as evaluated by AUROC. The same conclusion can also be drawn from the PR statistics.

**Table 1:** AUROC of our predictions.

	Ecoli1	Ecoli2	Yeast1	Yeast2	Yeast3
Size 10	0.928	0.912	0.949	0.747	0.714
Size 50	0.930	0.924	0.917	0.792	0.805
Size 100	0.948	0.960	0.915	0.856	0.783

**Table 2:** pAUROC of our predictions.

	Ecoli1	Ecoli2	Yeast1	Yeast2	Yeast3	Overall AUROC
Size 10	9.771e-07	2.629e-07	9.941e-07	2.931e-04	1.046e-03	9.523e-06
Size 50	2.396e-27	4.328e-31	1.477e-25	1.808e-21	1.386e-29	5.210e-27
Size 100	1.226e-52	5.876e-42	4.087e-70	5.755e-99	1.722e-92	3.112e-71

**Figure 1:**
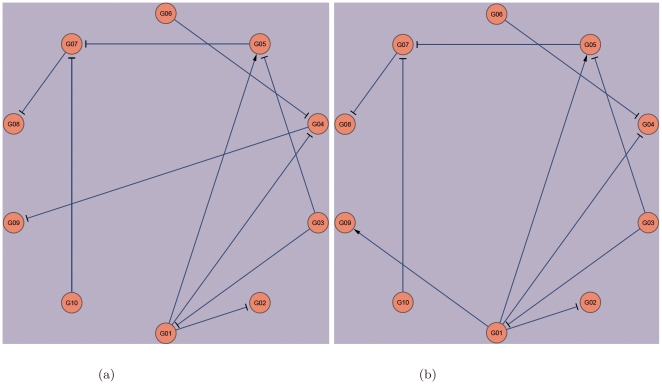
The Yeast1-size10 network. (a) The actual network. (b) Our top-10 predictions.

We notice that in some cases our first predictions are already very close to the actual network. Figure 1(a) shows the actual network of the Yeast1-size10 network, where an arrowhead represents an activation and a blunt-end represents a suppression. Figure 1(b) shows our top ten predictions. There is only one false positive (G01 activates G09) and one false negative (G04 suppresses G09). Interestingly, these two edges are tightly related. Since in the actual network G01 suppresses G04 and G04 suppresses G09, G01 can be viewed as indirectly activating G09. Our method thus correctly identified this relationship, yet it failed to distinguish between the direct and indirect regulation. We will discuss the issue of indirect regulation more in the next section. We also remark that although edge signs are not taken into account in the evaluation, our predicted edge signs are also consistent with the correct ones.

The overall scores are 5.124, 39.828, and 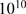, respectively, for the size 10, 50 and 100 networks. The unusual score for the size-100 network was due to a corresponding p-value too small to be represented numerically. As a comparison, the scores for the first runners-up are 3.821, 31.341 and 45.443, respectively. We hypothesize that the performance difference is at least partially attributed to our emphasis on the use of deletion data, as it appears that some other high-ranked teams put most of their concentration on building differential equation models from perturbation data (based on personal communications during the DREAM conference). To demonstrate the effectiveness of the noise models learned from deletion data, we analyze the number of predictions made in each batch, and the number of which are actually correct. The results for the size 10, 50 and 100 networks are shown in Table 3, Table 4 and Table 5, respectively. In the tables, each batch of predictions occupies a row, and the two columns for each network provide the total number or predictions in the batch and the number of correct ones among them, respectively. The last row shows the total numbers of all batches. The total number of predicted edges is equal to the total number of node pairs. For example, for the size-10 network, it is 10
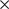
9 = 90. The total number of correct predictions is the number of edges in the actual network.

**Table 3:** Prediction accuracy per batch on the size 10 networks.

	Ecoli1		Ecoli2		Yeast1		Yeast2		Yeast3	
Batch	Predicted	Correct	Predicted	Correct	Predicted	Correct	Predicted	Correct	Predicted	Correct
1	11	7	16	12	11	9	13	9	12	8
2	6	1	4	0	5	0	5	1	5	4
3	0	0	1	1	3	0	1	0	1	0
4	5	1	8	0	7	0	4	2	4	0
5	4	0	8	1	6	0	10	3	5	1
6	1	1	0	0	0	0	0	0	0	0
7	63	1	53	1	58	1	57	10	63	9
Total	90	11	90	15	90	10	90	25	90	22

**Table 4:** Prediction accuracy per batch on the size 50 networks.

	Ecoli1		Ecoli2		Yeast1		Yeast2		Yeast3	
Batch	Predicted	Correct	Predicted	Correct	Predicted	Correct	Predicted	Correct	Predicted	Correct
1	96	52	133	69	145	57	176	83	201	100
2	76	2	85	1	80	8	87	12	102	16
3	77	0	78	1	69	1	56	1	64	2
4	196	0	153	1	185	1	156	5	113	3
5	178	1	169	1	167	2	177	6	149	2
6	5	0	16	0	9	0	11	0	6	0
7	1822	7	1816	9	1795	8	1787	53	1815	50
Total	2450	62	2450	82	2450	77	2450	160	2450	173

**Table 5:** Prediction accuracy per batch on the size 100 networks.

	Ecoli1		Ecoli2		Yeast1		Yeast2		Yeast3	
Batch	Predicted	Correct	Predicted	Correct	Predicted	Correct	Predicted	Correct	Predicted	Correct
1	410	101	377	108	483	118	656	257	710	302
2	387	11	319	1	317	20	282	22	311	31
3	162	0	198	0	129	0	145	3	135	3
4	650	0	685	1	575	2	604	12	638	13
5	683	1	656	2	746	3	739	10	667	24
6	53	0	72	0	82	2	67	0	59	2
7	7555	12	7593	7	7568	21	7407	85	7380	176
Total	9900	125	9900	119	9900	166	9900	389	9900	551

As hypothesized, the noise models learned from homozygous deletion data made very accurate predictions. In many cases, most actual edges were already predicted correctly in batch 1. Also, if an actual edge is not predicted in batch 1, it is also likely missed by subsequent batches. For instance, for the 173 actual edges in the Yeast3-size50 network, 100 are detected in batch 1, and among the remaining 73, only 21 are detected in batches 2 to 6.

While the above results suggest the importance of the noise models learned from homozygous data, it is still not clear whether these models are indeed more effective than the other models. It could still be the case that other models could also make the same predictions made in batch 1, just that as these predictions had already been covered in batch 1 that subsequent batches were not allowed to make the same predictions again. To verify if this was the case, we swapped the order of the first two batches for the size 10 networks, so that the first batch is composed of predictions made by the differential equation models and the second batch is composed of predictions made by the noise model learned from homozygous deletion data and not covered by the first batch. The results are shown in Table 6.

**Table 6:** Prediction of the first two batches on the size 10 networks when their orders are swapped.

	Ecoli1		Ecoli2		Yeast1		Yeast2		Yeast3	
Batch	Predicted	Correct	Predicted	Correct	Predicted	Correct	Predicted	Correct	Predicted	Correct
1	6	1	5	1	5	0	5	1	5	4
2	11	7	15	12	11	9	13	9	12	8

Comparing Table 6 and the first two batches of Table 3, it is seen that the number of predictions made by the models almost remained unchanged when the order of the two batches are swapped. In fact, by checking the predicted edges, it is observed that most predictions previously made by the noise model were not predicted by the differential equation models, even they were given the chance to freely make the predictions. Only one extra correct prediction could be made by the differential equation models for the Ecoli2 network. We remark that while the first two batches of predictions on Ecoli2-Size10 in the two tables have the same total number of predictions, they are not exactly the same predictions. This is possible because when the differential equation models are learned in batch 1, all data points are considered; but when they are learned in batch 2, the deviation of each point from mean is computed excluding the regulation already predicted by the noise model in batch 1. As a result, it is possible to have one more correct prediction out of the 20 predictions in Table 6.

This analysis reveals two interesting observations. First, as the noise models learned from deletion data gave higher accuracy than the differential equation models, our decision to use the former to make the first batch predictions is justified. Second, while the differential equation models had a lower accuracy, they had some small contributions to the prediction accuracy as they made some unique correct predictions that were missed by the noise models. As discussed, these are probably indirect or more complex regulation events.

To evaluate quantitatively the importance of the differential equation models, we use hypergeometric distribution to compute the probability of having at least the observed number of correctly predicted regulation events in batches 2–6 by chance, given the total number of predictions in these batches. For example, for the Ecoli1-Size10 network, we compute the probability of having 3 correct predictions (in batches 2–6) out of the 4 missed by batch 1, when making 16 predictions out of 89 node pairs (see Table 3). The result is a p-value of 0.0247, which is statistically significant at the 0.05 level. The complete set of p-values is shown in Table 7.

**Table 7:** Probability of having at least the observed number of correctly predicted regulation events in batches 2–6 by chance, given the total number of predictions in these batches.

	Ecoli1	Ecoli2	Yeast1	Yeast2	Yeast3
Size-10	0.0247	0.1922	1	0.1925	0.0923
Size-50	0.4015	0.3036	0.0003	0.0273	0.0078
Size-100	0.0012	0.1670	0.0000	0.0000	0.0001

Overall, in about half of the cases, the predictions made in batches 2–6 are significantly better than random at the 0.05 level. We observe that for networks with a large portion of real edges missed by batch 1 (such as Yeast3-Size100), the predictions of batches 2–6 are more significant. Our results thus suggest that the two types of models, based on two different types of data, are potentially capable of complementing each other and make some orthogonal contributions to the overall predictions.

**Figure 2:**
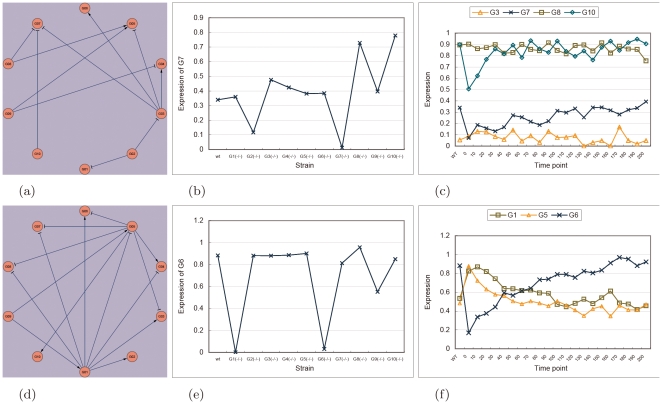
Two regulation events that were missed by the noise models but detected by the differential equation models. (a) The actual Ecoli1-size10 netowrk. (b) The homozygous deletion profile of G7 in the Ecoli1-size10 network. (c) A perturbation time series of G7 in the Ecoli1-size10 network. (d) The actual Ecoli2-size10 network. (e) A perturbation time series of G6 in the Ecoli2-size10 network. (f) A perturbation time series of G6 in the Ecoli2-size10 network.

Figure 2 shows two regulation events that can hardly be detected by deletion data alone. In the Ecoli1-size10 network, gene G7 is suppressed by G3, G8 and G10 (Figure 2(a)). Since G8 and G10 have high wild-type expression (Figure 2(c), wt), deleting G3 results in only a small increase in expression of G7 that can be difficult to detect (Figure 2(b)). In fact, this regulation event was missed in the first batch of prediction. On the other hand, by using a perturbation time series (Figure 2(c)), it can be seen that the expression of G7 increases even though the expression of G8 and G10 remain high. This suggests that the decrease in expression of G3, and thus its suppression to G7, could be the cause. The differential equation models were able to detect it in batch 2 of predictions (Table 3).

The second example is related to the Ecoli2-size10 network (Figure 2(d)). G6 is activated by G1 and suppressed by G5. G1 also suppresses G5. When G1 is expressed, the suppression of G6 by G5 is masked by the two functions of G1, which makes deleting G5 have a negligible effect on the expression of G6 (Figure 2(e)). The suppression of G6 by G5 was indeed missed by the first batch of predictions. From a perturbation time series (Figure 2(f)), the expression of G6 is observed to be anti-correlated with that of G1. This is unexpected, since anti-correlation is a phenomenon of a suppressor rather than an activator. The puzzle is solved by observing that G5 is also anti-correlated with G6, which leads our algorithm to correctly predict G5 as a suppressor of G6 in batch 3 (Table 3).

We have also briefly studied if the differential equation models can be improved by considering pairs of potential regulators instead of one single regulator at a time. For the five size-10 networks, we use the same algorithm as before, except that in batches 2–6 each model involves two potential regulators. The resulting AUC values for Ecoli1, Ecoli2, Yeast1, Yeast2 and Yeast3 are 0.887, 0.913, 0.943, 0.697 and 0.655 respectively. Comparing these numbers with those in Table 1, we notice that the accuracy is not improved by considering an extra potential regulator. We have tried several sets of parameter values, and the same conclusion is reached in all cases. We believe that the unsatisfactory results are due to over-fitting, as the number of parameters increases as we increase the number of potential regulators. This problem is especially serious for guided models, as they also involve other potential regulators detected in previous predictions.

## Discussion

Our prediction results demonstrate the advantage of combining multiple types of data. While the perturbation data allow the learning of differential equation models that could capture complex interactions in the regulatory network, deletion data also facilitate the detection of some simple interactions using only very basic noise models. As technological advancements are made rapidly, new data types are expected to come out from time to time. For method developers who try to improve existing prediction methods, besides deriving more advanced algorithms using the same data, it is also rewarding to investigate what kinds of information emerging data could provide, and how such information can be extracted to supplement existing methods.

As mentioned earlier, in this study we did not attempt to address the issue of indirect regulation. Indeed we observed that indirect regulation is one of the factors that confounded our method and caused it to make some wrong predictions. We expect that in a complete network with thousands of nodes, long regulation chains are prevalent and the problem of indirect regulation would be more serious. It is therefore interesting to see if filtering indirect regulation (for example by some existing techniques [15], [16]) could further improve the performance of the method. It would also be very useful to include other types of information in identifying direct regulation, such as direct protein-DNA binding data from ChIP-chip or ChIP-seq experiments [9].

In some previous work, more sophisticated noise models allowing for gene-specific and experiment-specific errors are proposed, with the aid of extra control experiments [21], [22]. When these control data are available, we believe the accuracy of our algorithm can be further improved by using such advanced noise models.

In this study, we adopt an unsupervised learning setting, in compliance with the setup of the DREAM3 challenge. For organisms with some known regulation edges as domain knowledge, they can be used as training examples to train a supervised learner, or be used to transform the existing method into a semi-supervised one [23]. For example, known examples can be used to setup p-value cutoffs in defining the potential regulation set 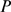 when learning the noise models. They can also help examine the validity of a particular differential equation model formulation, by checking if the squared errors of their best models are indeed smaller than average.

One issue that we have not touched on is the computational cost. Using a high-end cluster, our predictions for networks of size 10, 50 and 100 took about 2 minutes, 13 hours, and 78 hours, respectively. While there is room for optimizing our code, fitting the differential equation models intrinsically requires a lot of computational power. Given that most correct predictions are made by the noise models, which only took a tiny portion of the computational time, when working on complete networks it is possible to tradeoff some accuracy for much shorter running time. Alternatively, since a lot of the models are learned independently of each other, it is fairly straightforward to parallelize the computation and reduce the total running time by adding in extra machines.

## References

[1] Bonneau R, Reiss DJ, Shannon P, Facciotti M, Hood L (2006). The Inferelator: an algorithm for learning parsimonious regulatory networks from systems-biology data sets de novo.. Genome Biology.

[2] Gardner TS, di Bernardo D, Lorenz D, Collins JJ (2003). Inferring genetic networks and identifying compound mode of action via expression profiling.. Science.

[3] Mendes P, Sha W, Ye K (2003). Artificial gene networks for objective comparison of analysis algorithms.. Bioinformatics.

[4] Vu TT, Vohradsky J (2007). Nonlinear differential equation model for quantification of transcriptional regulation applied to microarray data of Saccharomyces cerevisiae.. Nucleic Acids Research.

[5] Cohen AA, Geva-Zatorsky N, Eden E, Frenkel-Morgenstern M, Issaeva I (2008). Dynamic proteomics of individual cancer cells in response to a drug.. Science.

[6] Giaever G, Chu AM, Ni L, Connelly C, Riles L (2002). Functional profiling of the saccharomyces cerevisiae genome.. Nature.

[7] Kamath RS, Fraser AG, Dong Y, Poulin G, Durbin R (2003). Systematic functional analysis of the Caenorhabditis elegans genome using RNAi.. Nature.

[8] Pe'er D, Regev A, Elidan G, Friedman N (2001). Inferring subnetworks from perturbed expression profiles.. Bioinformatics.

[9] Yeang CH, Ideker T, Jaakkola T (2004). Physical network models.. Journal of Computational Biology.

[10] Vaske CJ, House C, Luu T, Frank B, Yeang CH (2009). A factor graph nested effects model to identify networks from genetic perturbations.. PLoS Computational Biology.

[11] Markowetz F, Kostka D, Troyanskaya OG, Spang R (2007). Nested effects models for high-dimensional phenotyping screens.. Bioinformatics.

[12] The DREAM (dialogue for reverse engineering assessments and methods) project.. http://wiki.c2b2.columbia.edu/dream/index.php/Main_Page.

[13] Stolovitzky G, Prill RJ, Califano A (2009). Lessons from the DREAM2 challenges: A community effort to assess biological network inference.. Annals of the New York Academy of Sciences.

[14] Stolovitzky G, Monroe D, Califano A (2007). Dialogue on reverse-engineering assessment and methods: The DREAM of high-throughput pathway inference.. Annals of the New York Academy of Sciences.

[15] Wagner A (2004). Reconstructing pathways in large genetic networks from genetic perturbations.. Journal of Computational Biology.

[16] Rice JJ, Tu Y, Stolovitzky G (2004). Reconstructing biological networks using conditional correlation analysis.. Bioinformatics.

[17] Boyd S, Vandenberghe L (2004). Convex Optimization.

[18] Dahlquist G, Björck Å (2003). Numerical Methods.

[19] The DREAM3 challenges.. http://wiki.c2b2.columbia.edu/dream/index.php/The_DREAM3_Challenges.

[20] Marbach D, Schaffter T, Mattiussi C, Floreano D (2009). Generating realistic in silico gene networks for performance assessment of reverse engineering methods.. Journal of Computational Biology.

[21] Hughes TR, Marton MJ, Jones AR, Roberts CJ, Stoughton R (2000). Functional discovery via a compendium of expression profiles.. Cell.

[22] Hu Z, Killion PJ, Iyer VR (2007). Genetic reconstruction of a functional transcriptional regulatory network.. Nature Genetics.

[23] Chapelle O, Schölkopf B, Zien A (2006). Semi-Supervised Learning.

